# Biomarkers of selenium status in dogs

**DOI:** 10.1186/s12917-016-0639-2

**Published:** 2016-01-19

**Authors:** Mariëlle van Zelst, Myriam Hesta, Kerry Gray, Ruth Staunton, Gijs Du Laing, Geert P. J. Janssens

**Affiliations:** Department of Nutrition, Genetics and Ethology, Faculty of Veterinary Medicine, Ghent University, Merelbeke, Belgium; WALTHAM® Centre for Pet Nutrition, Waltham-on-the-Wolds, Leicestershire, UK; Department of Applied Analytical & Physical Chemistry, Faculty of Bioscience Engineering, Ghent University, Ghent, Belgium

**Keywords:** Selenium, Glutathione peroxidase, Thyroid hormones, Canine, mRNA expression, Urine

## Abstract

**Background:**

Inadequate dietary selenium (Se) intake in humans and animals can lead to long term health problems, such as cancer. In view of the owner’s desire for healthy longevity of companion animals, the impact of dietary Se provision on long term health effects warrants investigation. Little is currently known regards biomarkers, and rate of change of such biomarkers in relation to dietary selenium intake in dogs. In this study, selected biomarkers were assessed for their suitability to detect changes in dietary Se in adult dogs within eight weeks.

**Results:**

Twenty-four dogs were fed a semi-purified diet with an adequate amount of Se (46.1 μg/MJ) over an 8 week period. They were then divided into two groups. The first group remained on the adequate Se diet, the second were offered a semi-purified diet with a low Se concentration (6.5 μg/MJ; 31 % of the FEDIAF minimum) for 8 weeks. Weekly urine and blood was collected and hair growth measurements were performed. The urinary Se to creatinine ratio and serum Se concentration were significantly lower in dogs consuming the low Se diet from week 1 onwards, by 84 % (adequate 25.3, low 4.1) and 7 % (adequate 257 μg/L, low 238 μg/L) respectively. Serum and whole blood glutathione peroxidase were also significantly lower in dogs consuming the low Se diet from weeks 6 and 8 respectively. None of the other biomarkers (mRNA expression and serum copper, creatine kinase, triiodothyronine:thyroxine ratio and hair growth) responded significantly to the low Se diet over the 8 week period.

**Conclusions:**

This study demonstrated that urinary Se to creatinine ratio, serum Se and serum and whole blood glutathione peroxidase can be used as biomarkers of selenium status in dogs. Urinary Se to creatinine ratio and serum Se concentrations responded faster to decreased dietary Se than the other parameters. This makes these biomarkers candidates for early screening of long term effects of dietary Se provision on canine health.

## Background

Selenium (Se) is an essential trace element in dogs [1]. It is involved in many aspects of canine physiology, such as anti-oxidant protection [2], thyroid hormone metabolism [3], and immune function [4]. Although no naturally occurring clinical cases of Se deficiency or toxicity have been reported in dogs [1], an inadequate Se status is associated with long term health effects, such as calcium oxalate calculi [5, 6] and cancer [7, 8] formation.

Considering the importance dog owners place on the healthy longevity of their pets [9, 10], the health effects of dietary Se intake should be studied in more detail. Measuring only the dietary Se content is not sufficient to study effects of Se on metabolism as the bioactivity of Se, defined as the amount of dietary Se that can be incorporated into selenoproteins such as glutathione peroxidase (GPx), is affected by many factors [11]. Long term studies are costly and difficult to perform, and therefore biomarkers are needed which can identify Se-induced changes in metabolism at an early stage.

There is currently no literature on specific and sensitive biomarkers of dietary Se intake in dogs, which confounds the accurate assessment of Se status. The main tissues and body fluids for minimally-invasive measurement of Se concentration are whole blood, plasma, serum, erythrocytes, urine, hair, and nails [12]. In this study biomarkers were selected to assess their sensitivity to a manipulation of dietary Se concentration in adult dogs with an adequate Se status.

Glutathione peroxidase (GPx) is the biomarker most often measured to estimate Se bioactivity [13–15]. GPx is a selenoprotein that acts as an anti-oxidant [16] and is currently used as a proxy of selenium status, although has never been verified as the gold standard. GPx measurements in chicks receiving different concentrations of Se supplement [17] was used to inform the existing European minimum recommendation for the Se concentration in dog foods, which is 17.9 μg/MJ [18].

Secondly, Levander et al. [19] reported that urinary Se excretion in humans consuming a low Se diet (intake of approximately 35 μg/day) stabilised after 12 days. In cats, urinary Se concentrations increased dose-dependently within 2 days of Se supplementation with either sodium selenite or Se yeast at 1.5 and 2.0 mg Se/kg dry matter (DM) compared to 0.45 and 1.0 mg Se/kg DM [20].

The mRNA expression of one of the isoforms of GPx (GPx1) from liver tissue has also been reported to be a biomarker of Se status in rats [21, 22], together with selenoprotein H (SelH), selenoprotein W (SepW1), thioredoxin reductase 1 (TrxRd1), thioredoxin reductase 2 (TrxRd2), iodothyronine deiodinase 1 (DIO1), selenoprotein K (SelK), selenoprotein T (SelT), and 15 kDa selenoprotein (Sep15) [21, 22]. Although selenoprotein P is the most abundant selenoprotein [23] and is important in the transport of Se throughout the body [24], its mRNA has been shown to not be significantly down-regulated in Se deficiency [25].

Data from rats indicates that mRNA from whole blood can be used to measure mRNA biomarkers for Se status [26]. RNA isolated from whole blood for the determination of GPx1 mRNA was expressed at levels comparable to the levels found in liver, kidney and heart mRNA [26]. The expression of GPx1 mRNA in rats on a Se deficient torula yeast diet (0.007 μg Se/g diet) was only 14 % of the GPx1 mRNA expression in rats on a diet supplemented with 0.2 μg sodium selenite/g diet, which was comparable to the fall in liver mRNA [26].

Additionally, hair growth can be included as a biomarker because it has been reported to be reduced in beagles consuming a dietary Se concentration of 0.09 mg/kg DM (= approximately 5.5 μg/MJ) after 11 weeks [27]. Other indirect measures of Se status, such as serum creatine kinase (CK) and the thyroid hormones triiodothyronine (T3) and thyroxine (T4) were also included. Se is involved in the conversion of T4 into the active form T3 [28] and the T3:T4 ratio has been reported to decrease in puppies [29] and kittens [30] fed low compared to adequate Se concentrations. Finally, serum CK was increased in piglets fed either a diet containing no Se, although vitamin E levels were also manipulated in these studies [31].

## Methods

### Study design

A longitudinal, controlled and blinded study was performed using 24 adult Labrador retrievers. The dogs were assigned into 2 groups of 12 dogs, with age, gender and hair colour as blocking criteria. To ensure an adequate and stable selenium status in both groups at the start of the experimental period, the dogs received a Se adequate diet for 8 weeks before the experimental period. During this adaptation period, they were sampled three times (at weeks 2, 5 and 8) for baseline levels of blood and urine parameters and weekly hair growth measurements were performed. After the adaptation period, one group continued to receive the adequate Se diet and the other group received a diet with a low Se concentration for further 8 weeks. Weekly blood and urine samples were taken. In addition, weekly hair growth was measured. This study was approved by the WALTHAM^®^ Centre for Pet Nutrition Animal Welfare and Ethical Review Body and was conducted under UK Home Office Project Licence authorisation.

### Dogs

Twenty-four adult Labrador retrievers (14 female and 10 male) were selected for this study. All Labradors used in this study were bred at WALTHAM or sourced from Home Office approved breeders for research purposes. The average age, body weight (BW) and energy intake in kJ/kg metabolic body weight (BW^0.75^) per dog group are shown in Table 1. All dogs had an ideal body condition score (BCS) of D on the S.H.A.P.E™ BCS-scale [32]. Body weight and BCS was recorded before the start of the adaptation period and thereafter every week. The dogs were fed individually, once a day at 8:30 h, to energy requirements in order to maintain BW and BCS. They had access to fresh drinking water at all times. Food intake was also recorded throughout the study. Dogs were housed indoors in triplets with others of the same diet group with continuous access to an outside pen and access to an outside paddock during the day.

**Table 1:** Gender^a^, age, body weight and energy intake of the study dogs per dog group

	Number	♂	♀	Age (years)			Body weight (kg)			Energy intake (kJ/kg BW^0.75^)		
				mean	min	max	mean	min	max	mean	min	max
Group A	12	6	6	4.6	2.4	6.3	26.7	23.3	30.6	471	361	611
Group B	12	4	8	4.1	2.4	6.3	26.1	22.6	31.2	424	346	525

kJ, kilojoule; BW^0.75^, metabolic body weight; n, number of dogs; ♂, male; ♀, female; min, minimum; max, maximum

^a^All dogs were spayed/neutered

Both dog groups received the adequate Se diet in the 8-week pre-feeding period. Dog group A continued to receive the adequate Se diet in the 8-week experimental period and dog group B switched to the low Se diet

### Diets

Two semi-purified diets (Ssniff spezialdiäten GmbH, Soest, Germany) were used, one Se-adequate and one containing a low amount of Se (Table 2). Diets were formulated to be nutritionally complete for dogs with energy requirements of at least 397 kJ (95 kcal)/kg BW^0.75.^ Both diets had the same nutritional formulation, except for Se concentration. The Se adequate diet contained 46.1 μg Se/MJ and the low Se diet 6.5 μg Se/MJ, which is 31 % of the FEDIAF recommended minimum for dogs with an energy intake of 95 kcal/kg BW^0.75^ [18]. Se concentration from the ingredients was approximately 0.75 μg/MJ. Supplementary Se was in the form of sodium selenite.

**Table 2:** Analysed^a^ chemical composition of two semi-purified diets with an adequate or low selenium concentration

Component (g/MJ, except where specified)	Adequate Se diet	Low Se diet
Dry matter (g/100 g as is)	92.8	92.7
Crude protein	14.9	14.9
Crude fat	5.8	5.8
Crude fibre	1.6	1.5
Nitrogen free extract^b^	35.5	35.4
Starch	13.6	13.1
Crude ash	2.5	2.4
Metabolisable energy (MJ/kg DM)^c^	16.6	16.7
*Amino acids*		
Arginine	0.38	0.40
Histidine	0.21	0.21
Isoleucine	0.38	0.37
Leucine	0.68	0.68
Lysine	0.36	0.38
Methionine	0.32	0.36
Cysteine	0.36	0.36
Phenylalanine	0.45	0.47
Tyrosine	0.23	0.23
Threonine	0.47	0.47
Tryptophan	0.14	0.14
Valine	0.51	0.52
*Fatty acids*		
Linoleic acid	1.35	0.96
α-Linolenic acid (mg/MJ ME)	91	113
Arachidonic acid (mg/MJ ME)	22.6	9.3
EPA & DHA (mg/MJ ME)	6.9	6.5
*Minerals* (mg/MJ ME)		
Potassium	416	414
Calcium	383	388
Phosphorus	299	304
Chloride	123	110
Sodium	97	91
Magnesium	58.4	58.2
Zinc	6.5	6.5
Iron	3.4	3.9
Copper	0.48	0.68
Manganese	0.73	0.57
Iodine	0.08	0.08
Selenium (μg/MJ ME)	46.1	6.5
*Vitamins* (mg/MJ ME)		
Choline	148	146
Biotin	46.1	39.3
Niacin	5.5	4.9
Pantothenic acid	1.8	2.0
d-α-tocopherol	1.8	1.8
Riboflavin	0.45	0.44
Thiamin	0.24	0.27
Pyridoxine	0.14	0.22
Retinol	0.16	0.16
Menadion (μg/MJ ME)	71.4	90.6
Folic acid (μg/MJ ME)	53.2	45.2
Cyanocobalamin (μg/MJ ME)	35.6	37.5
Cholecalciferol (μg/MJ ME)	1.13	0.94

*MJ* megajoule, *ME* metabolisable energy, *EPA* Eicosapentaenoic acid, *DHA* docosahexaenoic acid

^a^All analyses were performed by Eurofins Food Testing, Wolverhampton, UK

^b^Calculated by substracting the amount (as g/100 g as is) of crude fat, protein, ash and fibre from the percentage dry matter and dividing this by the metabolisable energy concentration (MJ/kg as is)

^c^Calculated using predictive equations for ME (NRC, 2006)

Both dog groups received the adequate Se diet in the 8-week pre-feeding period. Dog group A continued to receive the adequate Se diet in the 8-week experimental period and dog group B switched to the low Se diet

### Blood samples

At weeks 2, 5 and 8 of the pre-feed period and weekly during the experimental period, blood samples (10.5 ml) were taken from each dog by jugular venipuncture using a 21 gauge needle. Blood was collected into a 2.5 ml PAXgene tube, 2 Microvette^®^ 500 μl lithium-heparin tubes, one 300 μl fluoride-heparin tube, one 200 μl Tri-Kalium-EDTA tube and two 4 ml Vacuette^®^ z serum clot activator tubes (one of them filled with only 2.5 ml of blood). The PAXgene tubes were incubated at room temperature for 2 h and stored at −20 °C for mRNA expression analysis. One of the lithium-heparin tubes was immediately stored at −80 °C and used for whole blood glutathione peroxidase (GPx) analysis at the end of the study. The other heparin tubes were centrifuged (accuSpin™ Micro R, Fisher Scientific™, Pittsburgh, PA, USA) immediately after collection at 1680 × g and 4 °C for 10 min. Lithium-heparin plasma was analysed for general biochemistry parameters and fluoride-heparin plasma for glucose (Spectrophotometry, Olympus AU400). EDTA tubes were placed on a roller at room temperature until analysis for haematology parameters (Orphée Mythic 18 Vet analyser) within 4 h of collection. Serum tubes were incubated for 30 min on ice and then centrifuged (Sigma 6 K15, rotor 11150, cups 13550, Sigma GmbH, Osterode am Harz, Germany) for 10 min. at 2000 × g and 4 °C. Serum samples were divided into Eppendorfs and stored at −80 °C for analysis of GPx, Se, triiodothyronine (T3), thyroxine (T4), copper (Cu) and creatine kinase (CK) at the end of the study.

### Urine samples

Free catch urine was collected weekly after feeding (between 8:30 and 16:00 h), using a Uripet urine collection device (Rocket Medical plc. Watford, England). From all urine samples, 1 ml was stored in an Eppendorf at −80 °C and analysed for creatinine (CT) within one month after sampling (IDEXX laboratories, UK). The rest of the samples were stored at −20 °C and analysed for total Se content (ICP-MS, PerkinElmer DRC-e, Waltham, USA).

### Hair growth measurements

Hair growth was measured according to a method adapted from Yu et al. [27]. Due to practical issues only chocolate and black Labradors were used for the hair growth measurements (5 black, 1 chocolate for the adequate Se diet and 6 black, 1 chocolate for the low Se diet). At the start of the pre-feed and experimental period, an area in the groin of approximately 5 × 5 cm was shaved with a 40 mm blade (Andis Super AGR+ cordless clipper; Andis Company Corporate, Sturtevant, Wisconsin, USA). Directly after shaving, the area was marked with a Duramark permanent marker. The shaved area was covered with a glass slide with a ruler attached to it, and a picture was taken using a Canon EOS 1000D digital camera with EF50mm f/2.5 compact-macro lens (Canon Inc., Melville, New York, USA) and Hoya PRO1 Digital Polarising Filter (HOYA Optics, Milpitas, California, USA). The glass slide was also used to flatten the hairs, when it had grown back in the following weeks to accurately measure them. Pictures were taken weekly during the pre-feed and experimental periods and were analysed with ImageJ analysing software [33]. Hair growth (mm/week) was measured as the difference of the average length of the hairs within the marked box between two consecutive pictures.

### Chemical analyses

Biochemistry and glucose analyses were carried out using spectrophotometry (Olympus AU400, Olympus Inc.) with Beckman Coulter reagents (Beckman Coulter Biomedical) within 20 mins of sampling. Haematology parameters were analysed using a Mythic 18 Vet analyser (Orphée S.A.). Serum and urine samples were prepared for total Se analyses with closed vessel microwave destruction as described in van Zelst et al. [34]. Se was analysed using inductively coupled plasma-MS (ICP-MS, Elan DRC-e, PerkinElmer), as described by Lavu et al. [35]. Urine CT was determined using a creatinine kit based on the Jaffe reaction (OSR6178, Beckman Coulter Biomedical, IDEXX Laboratories, London, UK).

Serum Cu and CK were analysed with the Randox copper and CK NAC-activated kit (Randox laboratories, London, UK), respectively, as per the manufacturer’s instructions. Thyroid hormones were analysed with canine T3 and T4 enzyme-linked immunosorbent assay (ELISA) kits (Cusabio, Wuhan, China). Whole blood GPx was analysed using the Ransel kit (Randox laboratories) and an Olympus AU400 spectrophotometer as per the manufacturer’s instructions. A 4-point calibration curve was used as control, with a 1:11, 1:21, 1:41 and 1:61 dilution. Whole blood and serum GPx analysis of the samples had an average coefficient of variation of 1.9 and 2.0 %, respectively. The 4-point calibration curve showed a recovery of 74.5, 87.1, 98.6, and 100.6 %, respectively.

mRNA expression was performed for the three baseline samples and the samples of weeks 2, 4, 6, and 8 of the experimental period. Total RNA, for mRNA expression, was isolated from the PAXgene tubes using the PAXgene blood RNA kit (Qiagen, Manchester, UK) as per the manufacturer’s instructions. The RNA was eluted in 2 × 40 μl of the elution buffer. Further DNase digestion of the RNA solution was carried out using RQ1 RNase-Free DNase (Promega, Southampton, UK) as per the manufacturer’s instructions with the sample incubated for 30 min at 37 °C. In order to remove the DNase and reaction buffer from the purified RNA, it was passed through the RNeasy Mini Kit using the RNA clean-up protocol and was eluted in 2 × 40 μl of elution buffer (10 mM Tris HCl, pH 8.4). The RNA concentration in the eluate was measured using the Qubit RNA Assay Kit (Invitrogen, Paisley, Scotland).

Primers and probes were designed using Primer3 [36] and M-Fold using the canine specific GenBank [37] sequences for GPx1, SelH, SepW1, TrxRd1, TrxRd2, DIO1, SelK, SelT, Sep15, tumor necrosis factor alpha (TNF-α), and nuclear factor kappa-light-chain-enhancer of activated B cells (NFκB) as described by Peters et al. [38]. The assays for the potential housekeeper genes were the same as described in Peters et al. [39].

Synthesis of complementary DNA (cDNA) was carried out with 750 ng of random hexamers using the ImProm-II Reverse Transcription System (Promega, Southampton, UK) and 750 ng of total RNA (as measured by the Qubit) in a final volume of 30 μl. All reactions were prepared according to the manufacturer’s instructions giving a final magnesium chloride concentration of 3 mM. The cDNA synthesis was carried out by mixing the RNA with the random primers in a reaction tube. Samples were heated to 70 °C for 5 min in the PTC-200 DNA engine (Bio-Rad Laboratories, Hemel Hempstead, UK) before cooling to 4 °C for 5 min. Tubes were placed in a cold block before addition of the reaction buffer, deoxynucleotides (dNTP’s), magnesium chloride, reverse transcriptase enzyme mix and water to make a total volume of 20 μl. Reverse transcription (RT) was undertaken by heating the samples to 25 °C for 5 min, 42 °C for 30 min and finally 75 °C for 10 min in the PTC-200 DNA engine (Bio-Rad Laboratories, Hemel Hempstead, UK). Duplicate RT reactions were performed for each RNA sample. All cDNAs were diluted 1:5 (v/v) for the genes of interest and housekeeper genes using EB Buffer (10 mM Tris–HCl pH-8.4, Qiagen, Manchester, UK) and then stored at −20 °C for future use. No template controls were performed by addition of nuclease-free water in place of RNA.

Quantitative polymerase chain reaction (qPCR) was performed using GoTaq Colourless Master Mix (Promega, Southampton, UK) as described by Van de Velde et al. [40]. The absence of genomic contamination of the RNA samples was confirmed prior to the RT reactions and none of the samples showed evidence of amplifiable genomic DNA with the succinate dehydrogenase complex, subunit A qPCR assay. One qPCR reaction was run for each RT repeat resulting in two threshold values (Ct) for each RNA sample. A mean Ct value was calculated for each sample using the two measured Ct values for each dog for each of the potential housekeeper genes. The mean Ct value was converted to a relative copy number value using the E^ΔCt^ method (E: reaction efficiency as determined from a standard curve) with ΔCt values calculated relative to the sample with the largest Ct (fewest gene copies). The geNorm visual basics for applications applet for Microsoft Excel was used to determine the most stable genes from the set of tested genes [41]. The three most stable housekeeper genes for the blood samples were beta-2-microglobulin (B2M), glyceraldehyde 3-phosphate dehydrogenase (GAPDH), and hypoxanthine phosphoribosyltransferase 1 (HPRT1). The primer and probe sequences of the genes of interest and these housekeeper genes are shown in Table 3.

**Table 3:** Primer and probe sequences used for positive control and qPCR assays

Gene	Forward primer	Reverse primer	Probe	Product size
GPx1	GTTCGGGCATCAGGAAAAC	TTCACCTCGCACTTCTCAAA	AAGTTGGGCTCGAACCCGCC	114
SelH	GGAGCTTTGGACTGGGATT	CTTCTGCCCAAACACCCTAC	CTTGAGTTTGCGTGGGGGCC	113
SepW1	GGCTACAAGTCCAAGTACCTTCAG	CCTCTCTTCTTGGAGTGAACCA	AAAGAAGCCGGTGGCCTGGG	148
TrxRd1	GTAGCAATCCAGGCAGGAAG	CAGGCACCATATTCCAAAGG	TGGCTCAGAGGCTTTATGCTGGC	116
TrxRd2	TTTATGCCATCGGAGACGTAG	CCATTATAGCCGTGGGTGTC	AGAGGGGCGGCCCGAGCT	60
DIO1	GAGGCTCTGGGTCCTCTTG	CCACGATGTGTTGCTTGACT	AGGTGGCCGTGGGCAAAGTG	95
SelK	AGGCTATGGAAACTCCTCTGATT	TTAATTCGACCCATTCTTCTGG	TGATGGAAGAGGGCCACCAGG	84
SelT	CGCTGCTCAAGTTCCAGATT	CCGCATGTACTCCTCAAACA	TGTGTTTCCTGAGGTTATAGGCGGG	65
Sep15	GGTCCTTCAAGCGGTGTCT	CAAGTTGCTGGAGAAGCCTAA	CGGGGCAGAGTTTTCATCAGAAGC	79
TNF-α	CATGTGCTCCTCACCCACAC	AGGGCTCTTGATGGCAGAGA	CGCTTCGCCGTCTCCTACCA	84
NFκB1	TGAGGATGGGATCTGCACT	CTCTGTCATTCGTGCTTCCA	TGGTCGGCTTTGCAAACCTGGG	124
B2M	ACGGAAAGGAGATGAAAGCA	CCTGCTCATTGGGAGTGAA	AGACCTGTCTTTCAGCAAGGACTGGACC	99
GAPDH	TCAACGGATTTGGCCGTATTGG	TGAAGGGGTCATTGATGGCG	CAGGGCTGCTTTTAACTCTGGCAAAGTGGA	90
HPRT1	CACTGGGAAAACAATGCAGA	ACAAAGTCAGGTTTATAGCCAACA	TGCTGGTGAAAAGGACCCCTCG	123

*qPCR* quantitative polymerase chain reaction, *GPx1* glutathione peroxidase 1, *SelH* selenoprotein H, *SepW1* selenoprotein W, *TrxRd1* thioredoxin reductase 1, *TrxRd2* thioredoxin reductase 2, *DIO1* iodothyronine deiodinase 1, *SelK* selenoprotein K, *SelT* selenoprotein T, *Sep15* 15 kDa selenoprotein, *TNF-α* tumor necrosis factor alpha, *NFκB1* nuclear factor kappa-light-chain-enhancer of activated B cells, *B2M* beta-2-microglobulin, *GAPDH* glyceraldehyde 3-phosphate dehydrogenase, *HPRT1* hypoxanthine phosphoribosyltransferase 1

Relative copy number expression values were calculated for each sample and normalised against the housekeeper gene results using the qBase applet for Microsoft Excel which employs the methodology described by Vandesompele et al. [41]. The sample with the fewest gene copies (latest Ct value) is given a relative copy number of 1 and all other samples are given values relative to this sample.

In order to assess reaction efficiency of the newly designed assays, a set of primers were designed for the gene target to amplify a larger fragment, which included the portion amplified by the qPCR assay. These assays were tested against a cDNA obtained from RNA extracted from canine blood. Products were separated by 2 % agarose gel electrophoresis, purified by NucleoSpin Extract II kit (Macherey-Nagel, Düren, Germany) and then quantified by QBit (Invitrogen, Paisley, Scotland). The number of copies per μl of purified product was calculated and then a 1:10 dilution series from 10^7^ to 1 copy per qPCR was analysed in duplicate using the qPCR assay and the reaction efficiency calculated using the MxPro software.

### Statistical analyses

The two primary parameters were whole blood GPx and urinary Se:CT ratio. The secondary parameters in this study were: Se intake, serum Se, serum GPx, serum CK, serum T3:T4 ratio, serum Cu relative to Cu intake, hair growth, and mRNA expression for GPx1, SelH, SepW1, TrxRd1, TrxRd2, DIO1, SelK, SelT, Sep15, TNF-α, and NFκB.

The average of the three baseline measurements were analysed between dog groups with two-sample Student’s t-tests. Using the entire dataset,each parameter was analysed univariately with a linear mixed effects model in R version 3.1.1 using the nlme package (R Foundation for Statistical Computing, Vienna, Austria). The fixed effects were diet, week, diet x week interaction, and baseline of the parameter (the average of the dogs’ three pre-feed measurements). The random effect was dog. The assumption of normality was assessed for each model by visual inspection of the residuals and urinary Se:CT, serum Cu and all mRNA parameters were log transformed. Between diet contrasts were applied in each week and between week contrasts were applied within each diet. Contrasts on the two primary parameters were Bonferroni corrected to an overall significance level of 5 % (i.e. threshold *p*-value of 0.025).

## Results

Se intake during the pre-feed period was similar in the two experimental groups (Group A: average 22.9 μg/kg BW^0.75^, range 19.0–29.5 μg/kg BW^0.75^, Group B: average 20.6 μg/kg BW^0.75^, range 16.8–25.2 μg/kg BW^0.75^). During the experimental period, average Se intake for the dogs on the adequate Se diet was 21.7 μg/kg BW^0.75^ (range 16.6–28.2 μg/kg BW^0.75^) and for the dogs on the low Se diet was 2.7 μg/kg BW^0.75^ (range 2.2–3.4 μg/kg BW^0.75^) which was significantly different comparing each week (*p* < 0.001). There were no significant differences between the groups for any of the parameters at baseline, except for the serum Cu:Cu intake ratio (*p* = 0.033).

**Fig. 1:**
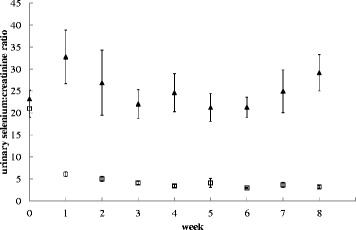
Urinary selenium to creatinine ratio of dogs fed a low or adequate selenium diet. The low selenium diet contained 6.5 μg/MJ and the adequate selenium diet 46.1 μg/MJ. Black triangles are dogs on the adequate selenium diet, open squares are dogs on the low selenium diet. Values at week zero indicate the average baseline values. Symbols represent the means and error bars indicate their standard errors, based on the raw data

**Fig. 2:**
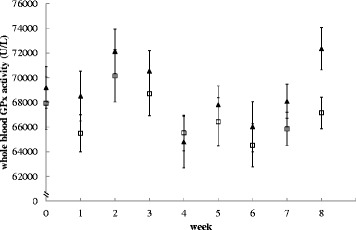
Glutathione peroxidase activity (U/L) in whole blood of dogs fed a low or adequate selenium diet. The low selenium diet contained 6.5 μg/MJ and the adequate selenium diet 46.1 μg/MJ. Black triangles are dogs on the adequate selenium diet, open squares are dogs on the low selenium diet. Values at week zero indicate the average baseline values. Symbols represent the means and error bars indicate their standard errors, based on the raw data

**Fig. 3:**
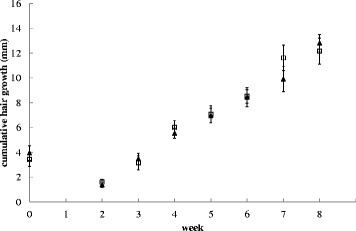
Cumulative hair growth (mm) of dogs fed a low or adequate selenium diet. Hair growth was measured weekly in the groin of Labrador retrievers fed a diet containing a low (6.5 μg/MJ) or adequate (46.1 μg/MJ) concentration of selenium. Black triangles are dogs on the adequate selenium diet, open squares are dogs on the low selenium diet. Values at week zero indicate the average baseline values at weeks 2, 5 and 8 of the pre-feed period, where hair had been removed at week 0. Symbols represent the means and error bars indicate their standard errors, based on the raw data

Urinary Se:CT ratio significantly decreased in dogs on the low Se diet from week 1 onwards and on average decreased by 84 % (Fig. 1). Whole blood GPx activity was significantly lower (by 7 %) in dogs fed the low Se diet at week 8 (Fig. 2). Cumulative hair growth results are shown in Fig. 3 and no differences between the diets were found. An overview of the results of all serum measurements is given in Table 4. Serum Se significantly decreased by 7 % in dogs fed the low compared to the adequate Se diet from week 1 onwards. A significant difference in serum GPx was detected from week 6 (6 % mean decrease from first significance).

**Table 4:** Serum biomarker concentrations in dogs fed a diet with a low or adequate selenium concentration

Diet	Week	Adequate-Se			Low-Se			*p*-value
		n	mean	SE	n	mean	SE	
Serum Se (μg/L)	0	12	263	5.3	12	263	4.7	0.987
	1	12	271	5.8	11	252	6.1	0.030
	2	12	263	3.6	12	245	6.0	0.029
	3	12	266	4.5	12	249	6.1	0.050
	4	12	261	4.5	12	236	3.5	0.000
	5	12	247	5.1	12	240	8.3	0.853
	6	12	248	4.6	12	230	6.4	0.024
	7	11	254	5.8	12	230	5.7	0.000
	8	12	243	4.7	12	224	4.1	0.013
Serum GPx (U/L)	0	12	14884	383	12	15027	323	0.778
	1	12	14754	355	11	14175	251	0.228
	2	12	15274	353	12	14563	380	0.101
	3	12	14726	309	12	14066	296	0.143
	4	12	14215	315	12	13644	304	0.250
	5	12	14421	304	12	14330	255	0.998
	6	12	14968	287	12	14007	313	0.012
	7	11	15052	349	12	14459	232	0.232
	8	12	15755	375	12	14579	271	0.001
Serum CK (U/L)	0	12	76	5.6	12	75	6.1	0.912
	1	10	79	6.3	10	75	5.2	1.000
	2	12	73	7.2	12	72	6.7	1.000
	3	12	84	10.7	11	75	5.9	0.965
	4	12	61	7.7	12	58	3.3	1.000
	5	12	45	4.4	12	54	5.2	0.837
	6	12	60	8.8	12	51	4.6	0.858
	7	11	52	3.8	11	56	4.5	1.000
	8	12	52	2.9	12	54	4.0	1.000
Serum T3:T4	0	12	0.031	0.001	12	0.029	0.001	0.093
	1	11	0.031	0.001	11	0.029	0.001	0.998
	2	12	0.031	0.001	12	0.031	0.001	0.987
	3	12	0.034	0.001	11	0.033	0.001	1.000
	4	12	0.038	0.001	12	0.033	0.001	0.144
	5	12	0.034	0.001	12	0.033	0.001	0.999
	6	12	0.034	0.001	12	0.031	0.001	0.900
	7	11	0.035	0.001	12	0.033	0.001	0.997
	8	12	0.036	0.001	12	0.035	0.001	0.996
Serum Cu:Cu intake	0	12	15.8	1.0	12	19.3	1.2	0.033
	1	7	32.1	2.5	8	22.8	3.5	0.985
	2	8	21.4	1.5	11	18.2	1.0	1.000
	3	12	23.2	1.6	10	20.0	1.0	0.944
	4	11	26.7	2.2	12	20.2	1.3	0.919
	5	12	17.4	0.8	12	14.8	0.7	0.999
	6	11	16.4	1.0	10	13.5	0.7	1.000
	7	8	17.5	1.0	6	13.1	1.0	1.000
	8	12	19.6	1.3	11	15.4	0.7	1.000

*Se* selenium, *n* number of dogs, *SE* standard error, *GPx* glutathione peroxidase, *CK* creatine kinase, *T3* triiodothyronine, *T4* thyroxine, *Cu* copper

Values at weeks zero indicate the average baseline values. Means and standard errors are based on the raw data

None of the mRNA measures were significantly changed by the consumption of the low Se diet (see Table 5). Interestingly, there was an upregulation in both groups in mRNA expression of SelK, SelT, and Sep15 during the second half of the experimental period (weeks 2 & 4 vs. weeks 6 & 8) and a downregulation in the expression of SelH, TrxRd1, TrxRd2, TNF-α, and NFκB (*p* < 0.05).

**Table 5:** Relative^a^ mRNA expression in dogs fed a diet with a low or adequate selenium concentration

Diet	Week	adequate-Se			Low-Se			*p*-value
		n	mean	SE	n	mean	SE	
GPx1	0	12	5.52	0.90	12	6.29	1.17	0.611
	2	12	5.93^a^	1.15	12	6.03^a^	1.35	0.956
	4	12	4.80^a^	0.77	12	4.62^a^	0.59	1.000
	6	12	4.42^a^	0.49	12	5.92^a^	1.24	0.950
	8	12	5.30^a^	0.48	12	6.27^a^	1.28	0.992
SelH	0	12	2.68	0.19	12	3.12	0.23	0.154
	2	12	3.58^a^	0.36	12	4.08^a^	0.26	0.972
	4	12	3.05^a^	0.29	12	3.38^a^	0.46	0.993
	6	12	2.20^b^	0.27	12	2.37^b^	0.19	1.000
	8	12	2.27^b^	0.36	12	2.20^b^	0.18	0.954
SepW1	0	12	2.16	0.24	12	2.62	0.35	0.286
	2	12	2.41^a^	0.31	12	2.41^a^	0.26	0.870
	4	12	2.48^a^	0.41	12	2.41^a^	0.44	0.581
	6	12	2.07^a^	0.26	12	2.05^a^	0.20	0.868
	8	12	2.61^a^	0.72	12	2.03^a^	0.30	0.468
TrxRd1	0	12	2.00	0.09	12	1.96	0.06	0.721
	2	12	5.54^a^	0.23	12	4.77^a^	0.38	0.259
	4	12	3.97^b^	0.26	12	3.89^a^	0.37	0.995
	6	12	1.89^bc^	0.15	12	1.67^b^	0.11	0.681
	8	12	1.64^bc^	0.09	12	1.65^b^	0.15	1.000
TrxRd2	0	12	2.86	0.19	12	3.01	0.20	0.615
	2	12	4.04^a^	0.40	12	3.84^a^	0.21	1.000
	4	12	3.75^a^	0.40	12	3.61^ac^	0.42	0.993
	6	12	2.25^b^	0.27	12	2.48^bc^	0.24	0.910
	8	12	3.17^ab^	0.64	12	2.31^b^	0.26	0.452
SelK	0	12	13.10	0.78	12	13.72	0.77	0.575
	2	12	2.78^a^	0.25	12	2.95^a^	0.23	0.998
	4	12	2.68^a^	0.25	12	2.80^a^	0.33	1.000
	6	12	11.42^b^	0.58	12	12.89^b^	1.17	0.982
	8	12	17.96^c^	2.01	12	15.91^b^	1.61	0.538
SelT	0	12	9.72	1.17	12	10.25	1.11	0.746
	2	12	4.27^a^	0.52	12	4.72^a^	0.52	0.921
	4	12	4.85^a^	0.63	12	5.05^a^	0.67	0.998
	6	12	7.12^b^	0.74	12	7.97^b^	1.07	1.000
	8	12	7.57^b^	0.86	12	7.92^b^	0.85	1.000
Sep15	0	12	2.37	0.15	12	2.47	0.19	0.693
	2	12	1.90^ac^	0.14	12	1.97^a^	0.11	0.996
	4	12	1.87^a^	0.20	12	1.71^a^	0.15	0.788
	6	12	2.44^b^	0.16	12	2.66^b^	0.21	0.962
	8	12	2.42^bc^	0.25	12	2.67^b^	0.31	0.953
TNF-α	0	12	3.28	0.30	12	3.54	0.38	0.597
	2	12	6.73^a^	0.91	12	7.03^a^	0.67	0.990
	4	12	6.73^a^	0.54	12	6.95^a^	0.66	1.000
	6	12	2.89^b^	0.40	12	3.31^b^	0.51	0.909
	8	12	3.29^b^	0.34	12	2.91^b^	0.29	0.681
NFκB	0	12	2.07	0.11	12	2.17	0.12	0.543
	2	12	5.18^a^	0.35	12	5.19^a^	0.21	1.000
	4	12	6.55^a^	0.34	12	6.26^a^	0.47	0.946
	6	12	1.72^b^	0.09	12	1.68^b^	0.13	0.982
	8	12	1.87^b^	0.16	12	1.71^b^	0.12	0.821

^a^Samples with the lowest number of gene copies for each gene of interest had a value of 1 with all other sample values relative to that. Values at weeks zero indicate the average baseline values. Weeks within one gene and diet with a common letter in superscript (mean column) do not significantly differ (*p* > 0.05). qPCR, quantitative polymerase chain reaction; Se, selenium; n, number of dogs; SE, standard error; GPx1, glutathione peroxidase 1; SelH, selenoprotein H; SepW1, selenoprotein W; TrxRd1, thioredoxin reductase 1; TrxRd2, thioredoxin reductase 2; DIO1, iodothyronine deiodinase 1; SelK, selenoprotein K; SelT, selenoprotein T; Sep15, 15 kDa selenoprotein; TNF-α, tumor necrosis factor alpha; NFκB1, nuclear factor kappa-light-chain-enhancer of activated B cells; B2M, beta-2-microglobulin; GAPDH, glyceraldehyde 3-phosphate dehydrogenase; HPRT1, hypoxanthine phosphoribosyltransferase 1

## Discussion

This study indicated that there was a potential difference in the reaction of biomarkers to changes in dietary Se concentration in dogs with an adequate Se status. Some of the measured biomarkers reacted within one week, whilst others did not significantly change within the 8 week study period. The estimated biomarker reaction time will be dependent on the power for each biomarker and so, more biomarkers may have been identified as significantly reacting if more dogs were included in the study. The number of dogs in this study (*n* = 24) was selected to ensure 80 % power to detect differences between the diets in the primary measures: urinary Se:CT and whole blood GPx. This is considered a realistic number to be repeated at other dog facilities, as larger numbers of dogs are rarely found in research facilities. This study did not evaluate the effects of Se on dog health, but does give an indication on which parameters may be used to assess Se associated long term health effects.

Urinary Se:CT ratio dramatically decreased within one week after changing to the low Se diet. Urine is the main route of excretion of excess Se [42, 43]. 24-h urinary Se concentrations have previously been demonstrated to correlate well with Se intake in humans [43, 44], cats [15, 20], and dogs [15] and this study demonstrated that the more practical single void sample, corrected for CT concentration, is a valuable indicator of dietary Se concentration in dogs. Urinary Se:CT ratio has previously been validated as a proxy measure of 24-h urinary Se excretion in humans [45] but has never been measured in diseased dogs (e.g. with cancer), so there is no dog specific estimate of a safe urinary Se:CT concentration. However, in humans there are indications that urinary Se reflects Se requirements, as excretion decreases in healthy children, pregnant women and in people with cancer, where it is likely that Se requirements are higher compared to healthy individuals [43]. This makes urinary Se:CT ratio an important biomarker to measure changes in dietary Se intake, but the safe minimum level of urinary Se:CT ratio that is associated with the prevention of long term health effects warrants further research.

Serum Se concentrations also decreased within one week after switching the dogs to the low Se diet. The average serum Se concentrations of the dogs in this study was 257 μg/L (range 213–310 μg/L) in the adequate and 238 μg/L (range 172–298 μg/L) in the low Se group, and fell within the range reported by Forrer et al. [46] (150–340 μg/L) for healthy dogs. However, dogs in the study of Forrer et al. were healthy at the time of sampling, but were not monitored during a longer period, so disease may have developed at a later stage. Pilarczyk et al. [47] found a significantly lower (*p* < 0.01) serum Se concentration in dogs with malignant neoplasm (range 103–265 μg/L) compared to healthy dogs (range 208–346 μg/L). This association reinforces the need to verify whether this relationship is causal.

GPx measurements were taken as an indicator of Se bioactivity. Se requirements are often based on GPx concentrations reaching a plateau [29, 48]. However, we do not know if optimal selenoprotein concentrations are desirable for optimal health. In humans, a blood Se concentration of 79–95 μg/L is considered sufficient to maximize GPx activity [48]. This seems to be higher in dogs, as the average serum Se concentration of dogs in the low Se group in the last week of the study was 224 μg/L, where both serum and whole blood GPx concentrations were significantly lower than in the adequate Se group, and thus not maximised.

In this study, inorganic Se (sodium selenite) was used as the primary source of Se. This can be converted to selenocysteine and incorporated into selenoproteins [49]. Selenomethionine cannot be synthesized from inorganic selenium, but may be non-specifically incorporated into body proteins [50], so using inorganic Se prevents selenomethionine mixing with the methionine pool and incorporation of selenomethionine into body proteins where it has no Se specific role [49]. The use of selenomethionine (organically-bound Se) would have likely resulted in a higher retention of Se in the body (i.e. lower excretion) without being bioactive, and a higher dietary Se requirement may thus apply for organic compared to inorganic Se species in order to maximize selenoprotein activity.

The body stores of Se may also have had an impact on all biomarkers measured in this study. In the first part of the study, Se may still have been present in body proteins and during protein turn-over may have been released to become available for selenoprotein incorporation (in this study measured as GPx), while in the last few weeks body stores of Se may have become depleted. It is likely that the amount of stored Se is animal species specific, as Todd et al. [15] found that cats retain less Se than dogs. This may be linked to the evolutionary “feast and famine” feeding approach of dogs, as described by Bosch et al. [51], in which dogs gorge feed, and during the famine stage, stored Se may be used for physiological processes.

Serum GPx, in this study, significantly reacted two weeks earlier to a change in dietary Se compared to whole blood GPx. Similar findings were reported in cats by Todd et al. [20]. Changes in plasma GPx were observed after feeding a diet containing 2, 1.43 and 0.98 mg sodium selenite/kg DM after 16, 32 and 24 days, respectively, compared to a basal diet containing 0.45 mg Se/kg DM. No changes in whole blood GPx were observed within the 32-day lasting study of Todd et al. [20]. This difference may simply be caused by the higher level and variation of GPx activity in whole blood compared to serum. GPx activity of dogs in whole blood is approximately five times higher than in serum. Another possible reasoning is that the surplus of tissue GPx is effluxed into the plasma, as indicated by the high correlation between tissue and plasma GPx concentrations [52]. The difference in reaction time between serum and whole blood GPx activity may also be explained by different isoforms of GPx. Plasma is known to contain mainly the plasma GPx (GPx3) enzyme [53], while whole blood may additionally contain other forms of GPx. However, the GPx activity measurement does not discriminate between various forms of GPx and it may be that the activity of serum GPx is more quickly affected by a reduction in dietary Se than other forms of GPx. In rats, it has been determined that plasma GPx3 activity is more highly regulated by dietary Se concentration than, for example, muscle GPx4 [54]. The same authors [54] have shown that although GPx1 mRNA expression was highly regulated by dietary Se concentration in rats, dietary Se requirements based on tissue (plasma, red blood cell, liver, kidney, and muscle) GPx activity were higher than when based on mRNA expression (i.e. mRNA expression plateaus at lower dietary Se concentrations). This is in accordance with the findings of the present study. The fact that GPx is affected, but the selenoprotein mRNA levels are not, indicates that the bioactive Se fraction of the low Se diet (total Se concentration: 6.5 μg/MJ or 0.11 mg/kg DM) is sufficient to maintain mRNA expression of the measured selenoproteins. Although mRNA expression is the basis of protein formation, the actual formation of the active selenoproteins were not maximized by the low Se diet. This may be due to effects on translation, like the structure of specific stem-loops which bind proteins [55]. Also, post-translational modifications (which affect the actual activity of the selenoproteins, e.g. protein folding, phosphorylation, etc.) may have played a role in the difference in reaction of GPx1 mRNA expression and GPx activity to a change in dietary Se concentration. Therefore, GPx activity may be a more reliable biomarker for Se status than the mRNA expression of selenoproteins. However, it may be that maximal selenoprotein activity is not necessary for the prevention of disease on the long term. Selenius et al. [56], for example, found that TrxRd1 mRNA expression was increased with increasing addition of sodium selenite concentrations (range 2.5–10 μM) to lung cancer cells, while TrxRd activity decreased at high selenite concentrations, which indicates the impairment of selenoprotein formation. Therefore, it is hypothesized that selenoprotein mRNA expression may be a useful biomarker in the detection of high dietary Se concentrations, but that GPx activity is more useful in the estimation of minimum Se requirements.

The fact that the SelK, SelT, and Sep15 mRNA markers were increased and SelH, TrxRd1, TrxRd2, TNF-α, and NFκB were decreased in the second half compared to the first half of the study, point towards an event of inflammation during the first part of the study, as TNF-α, and NFκB are inflammation markers. The study did not include induction of inflammation and there are no indications of such events during or before the study, all circumstances stayed the same. This reinforces the idea that other factors than dietary Se have more impact on the selenoprotein mRNA expression than dietary Se concentration.

The dietary Se concentration did not result in a significant difference in hair growth between the diets. Previous studies which have used hair growth as a marker of Se status [27] used beagles, so there may be a breed variation in hair growth. Although no literature on speed of hair growth between different breeds could be found, there is evidence for genetic differences in coat formation between breeds [57]. However, the most likely explanation for the lack of difference in hair growth is that the current study has used sodium selenite as Se source and in the study of Yu et al. [27], selenomethionine was supplemented. Methionine is often a limiting amino acid and selenomethionine can be non-specifically incorporated into body proteins such as hair [50]. Therefore, it may be that the decrease in hair growth in the study of Yu et al. is not due to a reduction in Se, but in methionine.

As there is no clear adequate range of dietary Se for adult dogs, the low Se diet may have contained sufficient Se (6.5 μg/MJ or 0.11 mg/kg DM) to maintain health. In this 8 week study it did not have a negative effect on thyroid hormone metabolism (T3:T4). The conversion of T4 into the active form T3 has been reported to increase with increasing dietary Se concentrations in puppies within a range of 0–0.52 mg/kg [29]. Also Cu is indicated to be involved in thyroid hormone metabolism, as a lower GPx activity and liver selenodeiodinase activity was found in Cu deficient rats compared to a control group [58]. These results indicate that the low Se diet is not deficient for thyroid hormone metabolism of adult dogs. Although the serum Cu:Cu intake ratio was significantly different between the groups at baseline, this is unlikely to have affected the results as including the baseline intake as a covariate in the analysis will normalize any differences between groups. Further studies are required to determine the long term health effects associated with a change in urinary Se:CT ratio, serum Se and serum GPx concentrations. This can be done by a retrospective study to determine adequate ranges of these markers and to establish which set of markers from our study is needed to provide a sufficient prediction of the risk of Se-induced disease. Consecutively, these ranges can be used in a study to determine the minimal Se requirement of dogs.

## Conclusion

There were variations in the reaction time of Se biomarkers to a reduction in dietary Se concentration in dogs. This study has demonstrated that urinary Se:CT ratio, serum Se, and serum GPx activity react most quickly. These may be useful biomarkers in future long term studies to evaluate the minimum requirements for optimal health in dogs.

## Abbreviations

B2M: beta-2-microglobulin
BCS: Body condition score
BW: Body weight
BW^0.75^: Metabolic body weight
cDNA: Complementary deoxyribonucleic acid
CK: Creatine kinase
Ct: Threshold value
CT: Creatinine
Cu: Copper
DIO: Iodothyronine deiodinase
DM: Dry matter
ELISA: Enzyme-linked immunosorbent assay
GPx: Glutathione peroxidase
GAPDH: Glyceraldehyde 3-phosphate dehydrogenase
HPRT1: Hypoxanthine phosphoribosyltransferase 1
MJ: Mega joule
mRNA: Messenger ribonucleic acid
NFκB: Nuclear factor kappa-light-chain-enhancer of activated B cells
qPCR: Quantitative polymerase chain reaction
RT: Reverse transcription
Se: Selenium
SeCys: Selenocysteine
SelH: Selenoprotein H
SelK: Selenoprotein K
SelT: Selenoprotein T
SeMet: Selenomethionine
Sep15: 15 kDa selenoprotein
SepW1: Selenoprotein W
T3: Triiodothyronine
T4: Thyroxine
TNF-α: Tumor necrosis factor alpha
TrxRd: Thioredoxin reductase
